# Literature-Based Discovery to Elucidate the Biological Links between Resistant Hypertension and COVID-19

**DOI:** 10.3390/biology12091269

**Published:** 2023-09-21

**Authors:** David Kartchner, Kevin McCoy, Janhvi Dubey, Dongyu Zhang, Kevin Zheng, Rushda Umrani, James J. Kim, Cassie S. Mitchell

**Affiliations:** 1 Laboratory for Pathology Dynamics, Georgia Institute of Technology and Emory University, Atlanta, GA 30332, USA; 2 Department of Biomedical Engineering, Georgia Institute of Technology and Emory University, Atlanta, GA 30332, USA; 3 College of Computing, Georgia Institute of Technology, Atlanta, GA 30332, USA; 4 Center for Machine Learning at Georgia Tech, Georgia Institute of Technology, Atlanta, GA 30332, USA

**Keywords:** COVID-19, SARS-CoV-2, resistant hypertension, cardiovascular disease, adverse event, text mining, knowledge graph, unsupervised machine learning, artificial intelligence

## Abstract

**Simple Summary:**

Given the prevalence of COVID-19 infection, assessment of sequelae is critical to public health. Recent studies revealed a higher incidence of resistant hypertension after COVID-19 recovery. Presently, there is limited data and ability to clinically ascribe mechanisms using traditional techniques. Literature-based discovery (LBD) leverages artificial intelligence to stitch together multi-scalar relationships from millions of journal articles. Identified related concepts are ranked according to their predicted relevance in ascribing the shared etiology of hypertension and COVID-19. The dominant LBD-identified ascribed physiology included: altered endocrine function, inflammation, lipid dysfunction, altered nerve input for blood pressure, and altered COVID-19 viral entry.

**Abstract:**

Multiple studies have reported new or exacerbated persistent or resistant hypertension in patients previously infected with COVID-19. We used literature-based discovery to identify and prioritize multi-scalar explanatory biology that relates resistant hypertension to COVID-19. Cross-domain text mining of 33+ million PubMed articles within a comprehensive knowledge graph was performed using SemNet 2.0. Unsupervised rank aggregation determined which concepts were most relevant utilizing the normalized HeteSim score. A series of simulations identified concepts directly related to COVID-19 and resistant hypertension or connected via one of three renin–angiotensin–aldosterone system hub nodes (mineralocorticoid receptor, epithelial sodium channel, angiotensin I receptor). The top-ranking concepts relating COVID-19 to resistant hypertension included: cGMP-dependent protein kinase II, MAP3K1, haspin, ral guanine nucleotide exchange factor, N-(3-Oxododecanoyl)-L-homoserine lactone, aspartic endopeptidases, metabotropic glutamate receptors, choline-phosphate cytidylyltransferase, protein tyrosine phosphatase, tat genes, MAP3K10, uridine kinase, dicer enzyme, CMD1B, USP17L2, FLNA, exportin 5, somatotropin releasing hormone, beta-melanocyte stimulating hormone, pegylated leptin, beta-lipoprotein, corticotropin, growth hormone-releasing peptide 2, pro-opiomelanocortin, alpha-melanocyte stimulating hormone, prolactin, thyroid hormone, poly-beta-hydroxybutyrate depolymerase, CR 1392, BCR-ABL fusion gene, high density lipoprotein sphingomyelin, pregnancy-associated murine protein 1, recQ4 helicase, immunoglobulin heavy chain variable domain, aglycotransferrin, host cell factor C1, ATP6V0D1, imipramine demethylase, TRIM40, H3C2 gene, COL1A1+COL1A2 gene, QARS gene, VPS54, TPM2, MPST, EXOSC2, ribosomal protein S10, TAP-144, gonadotropins, human gonadotropin releasing hormone 1, beta-lipotropin, octreotide, salmon calcitonin, des-n-octanoyl ghrelin, liraglutide, gastrins. Concepts were mapped to six physiological themes: altered endocrine function, 23.1%; inflammation or cytokine storm, 21.3%; lipid metabolism and atherosclerosis, 17.6%; sympathetic input to blood pressure regulation, 16.7%; altered entry of COVID-19 virus, 14.8%; and unknown, 6.5%.

## 1. Introduction

COVID-19 typically results in a mild–moderate respiratory illness. However, patients with underlying conditions or certain predispositions typically face more severe illness, and oftentimes, hospitalization [1]. Even during the height of the SARS-CoV-2 (i.e., COVID-19) pandemic, cardiovascular disease (CVD) had a greater mortality than the COVID-19 virus [2]. With more time to focus on the longer-term and broader effects of COVID-19 since the pandemic’s onset, recent literature indicates that cardiovascular complications are prevalent during and following COVID-19 infection [2]. Further, patient populations with pre-existing CVD or CVD risk factors experience worse disease outcomes [3,4]. Documented CVD adverse events during active COVID-19 infection and/or recovery include myocarditis, acute myocardial infarction, cardiomyopathy, arrythmias, and resistant hypertension [2].

There are many etiological questions that remain as to how COVID-19 intersects with CVD. The etiology of acute COVID-19 CVD adverse events like myocarditis [5], an inflammation of the heart valves often associated with viral infection, is more straightforward. However, the causes of chronic resistant hypertension after COVID-19 are less clear. Resistant hypertension is defined as abnormally high blood pressure that remains unrepressed under the treatment of at least three separate antihypertensive drugs [6]. Resistant hypertension is reported to be a significant predictor of fatalities in hospitalized COVID-19 patients compared to regulated hypertension [6,7]. While ample research has highlighted the hypertensive properties of COVID-19, little is known about the underlying mechanisms that explain potential interplay between COVID-19 and new onset or exacerbated resistant hypertension.

A recent clinical study reported that between 10% and 14% of adult COVID-19 patients will develop new onset or worsened hypertension after COVID-19 recovery, typically about 2 months after their initial COVID-19 infection [8]. Another large study with 45,398 COVID-19 patients and 13,864 influenza patients found that new-onset, persistent hypertension was 21% in COVID-19 compared to 16% with influenza at approximately 6 months after initial infection [9]. The study by Zhang and colleagues examined the odds ratio and 95% confidence interval from developing persistent hypertension after infection. Hospitalized patients with COVID-19 were 2.23 times ([95%, 1.48–3.54]; *p* < 0.001) more likely and non-hospitalized patients with COVID-19 were 1.52 ([95% CI, 1.22–1.90]; *p* < 0.01) times more likely to develop persistent hypertension compared to those with influenza [9]. These findings indicate that viral infection increases risk of hypertension, but the risk is particularly worse with COVID-19 compared to other infections. Another clinical study with 366 hospitalized COVID-19 patients founds hypertension was a sequela to infection; 190 patients with previous hypertension had a significantly increased level of angiotensin II, procalcitonin, and cTnl [10]. In addition to hypertension, other related sequela reported after COVID-19 infection include dyslipidemia and diabetes [11]. In short, multiple studies have identified the presence of existing and new onset persistent and/or resistant hypertension both during COVID-19 and several months after initial infection recovery.

Prior work has identified and linked COVID-19 with the angiotensin II-converting enzyme receptor (ACE2 receptor), which serves as the main entry point for viral infection [12]. Interactions of the SAR-CoV-2 virus with ACE2 receptor expression within the lungs leads to acute respiratory disease and other respiratory complications, such as difficulty breathing. Further, ACE2 expression has been documented across various cell types and organs outside the lungs. Thus, it is widely hypothesized that the severe and widespread impact of COVID-19 within the body is a direct result of viral interactions with ACE2 receptor expressing cells [1]. Notably, ACE2 is part of the renin–angiotensin–aldosterone system (RAAS), which is key for blood pressure regulation [13]. Renin, angiotensin, and aldosterone elevate arterial pressure in response to decreased renal blood pressure, salt delivery to the distal convoluted tubule, and beta-agonism [14]. Angiotensin-converting enzyme (ACE) inhibitors are a common therapy for treating hypertension. ACE inhibitors suppress an enzyme that is required to produce angiotensin II, which is associated with narrowing of blood vessels [13]. Epithelial sodium channels are another component of the RAAS pathway and are responsible for sodium reuptake. Epithelial sodium channels have also been shown to be affected by COVID-19 [15]. 

Presently, there has been little research performed to ascribe the potential causal links between COVID-19 and resistant hypertension. Most studies have examined clinical associations and not deeper, multi-scalar biology. Assessment of the etiology of resistant hypertension and COVID-19 warrants a cross-domain, interdisciplinary perspective. New advanced tools allow the scientific field to rapidly discover and publish data. There is great opportunity to leverage the current published literature to provide the cross-domain perspective necessary to answer complex questions that are not easily answered using more traditional techniques. Text mining is one of the few techniques that enables holistic examination of multi-scalar and multifactorial relationships across all domains. Literature-based discovery (LBD) has long held promise since it was first utilized to identify that fish oil, which is hypothesized to reduce blood viscosity, is a good therapy for Raynaud’s phenomenon [16]. Recent algorithmic advances have enabled more wide-scale utilization of LBD in biomedical science for drug repurposing, risk assessment, question answering, and research prioritization [17].

The presented work examines the relationship(s) between COVID-19 and resistant hypertension using LBD. Artificial-intelligence-based LBD enables examination of millions of journal articles to aggregate knowledge in a comprehensive, less biased manner compared to traditional systematic reviews. Due to the inherent limits of manual human review of articles, traditional systematic reviews comparatively include only a very tiny fraction of articles, typically around 100-200 articles, which are restricted to the immediate domain of interest instead of incorporating cross-domain connections. As such, traditional human-based systematic reviews are likely to result in a more narrow and unintentionally biased review. As such, traditional systematic reviews would most likely be presently inferior for exploring more distant, lesser understood etiological relationships between COVID-19 and resistant hypertension.

The SemNet family [18,19] of LBD tools extract semantic relationships from the text of all the articles in the PubMed database. Biomedical concepts (e.g., nodes) and relationships (e.g., edges) are stitched together into a comprehensive knowledge graph. Metapaths specify the pattern of relationships between user-specified biomedical concepts of interest, called target nodes, and related biomedical concepts in the graph, called source nodes [18]. The simplest metapath structure is concept → relationship → concept, such as COVID-19 → causes → hypertension. Unsupervised rank aggregation is used to compare metapaths and rank the importance of related source nodes using a relevance-based metric called a HeteSim score [18]. Degree-weighted path counts are utilized to minimize the occurrence of overly common metapaths from being predicted as more important simply because they are more prevalent in the literature [18,19].

LBD with the SemNet family of tools [18,19] has already succeeded in identifying and ranking both existing and novel or under-studied relationships. SemNet with link prediction was able to successfully forecast repurposed drugs for COVID-19 [20]. More than 40% of the predicted repurposed drugs for COVID-19 went on to be clinically validated as COVID-19-adjuvant therapies [21]. SemNet 2.0 [18] (the latest version of SemNet at the time of this writing) with cross-domain text mining was utilized to forecast long-term adverse events from chronic tyrosine kinase inhibitor therapy for chronic myeloid leukemia [22]. Additionally, SemNet 2.0 has been used to highlight the promise of antihistamines as a prioritized adjuvant therapy with levodopa derivatives in the treatment of Parkinson’s Disease [23]. In short, LBD with SemNet provides a comprehensive method for exploring and prioritizing biomedical concepts of interest for future experimental or clinical validation.

Here the SemNet 2.0 software [18] was utilized to query a knowledge graph of biomedical concepts generated from 33+ million PubMed articles. The algorithm identified and ranked relevant concepts that represent direct or indirect connections between COVID-19 and resistant hypertension. Furthermore, a unique cross-domain serial SemNet 2.0 simulation method [22] using identified hub nodes combed relationships from cardiology, neurology, oncology, and numerous other domains to unearth possible etiological connections that may otherwise go unseen. Much akin to hub node network analysis in bioinformatics [24], hub nodes are selected during cross-domain text mining to formulate a series of simulations that are more expansive and can extend analysis to lesser-studied relationships [22]. As such, cross-domain text mining and hub network analysis with SemNet 2.0 [18] identifies more distant or under-studied relationships than traditionally possible with LBD. In summary, cross-domain text mining and hub node analysis with SemNet 2.0 elucidated, synthesized, and prioritized key multi-scalar and multifactorial concepts that explain the connection(s) between COVID-19 and resistant hypertension.

## 2. Materials and Methods

LBD uses existing, published literature and extracts relationships from it. Data that are already published inform connections between concepts that have not yet been explicitly linked. LBD methods enable experts of the biomedical community to cross-link domains of work and navigate the expanse of data that are available in a more feasible way [25]. In this work, LBD, and specifically SemNet 2.0, was utilized to support the extraction and ranking of biological relationships between COVID-19 and resistant hypertension.

### 2.1. Overview of SemNet 2.0 Software for Literature-Based Discovery

SemNet is a heterogenous knowledge graph generated by text mining of 33+ million published journal articles from PubMed. The database contains subject–predicate–object triplets where the subject and object are biomedical concepts, and the predicate is a relationship between those concepts [18,19]. For example, a triplet within the data may be aldosterone → increases → blood pressure. SemNet 2.0 generates a heterogeneous information network where nodes represent biomedical concepts and edges represent the relationships between nodes. Nodes and edges are encoded using the Unified Medical Language System (UMLS) ontology [18,19].

Queries are performed on the graph to examine how concepts within the graph are interconnected to a set of specific target nodes. Target nodes are user-defined UMLS nodes. Target node selection is typically guided by domain expertise to answer a specific hypothesis. Query results consist of source nodes, which are nodes that share a connection with the target node(s). The list of nodes and edges that connect the target node to a source node is denoted as a metapath. The length of a metapath is a controllable parameter of a SemNet query. Other parameters include the following: (1) specifying returned UMLS source node types; (2) specifying the source node search depth. Examples of common UMLS node types include the following: gene or genome (GNGN), amino acid; peptide or protein (AAPP); hormone (HORM); pharmacological substance (PHSU); disease or syndrome (DSYN). The search depth specifies how many connections away from the target node the algorithm will search for connected source nodes [18,19]. Specifying the UMLS source node type(s) and simulation search depth makes the simulation computationally tractable and ensures the results best answer the question(s) posed by the SemNet 2.0 literature search.

SemNet queries return the list of ranked source nodes connected between target nodes. Each source node can have many metapaths connecting it back to the target node(s) [18]. SemNet ranks the returned source nodes using an unsupervised learning rank aggregation algorithm [18,19]. An unsupervised methodology looks for otherwise hidden patterns in unlabeled data. The revised unsupervised learning algorithm for rank aggregation (ULARA) algorithm in SemNet 2.0 uses a majority voting process that considers all metapaths and then predicts which ones are most relevant to the target [18]. The results of SemNet simulations include the returned source nodes from the queried knowledge graph and their corresponding HeteSim relevance scores. The HeteSim score measures the relevance of the source nodes by evaluating the metapaths between the target node and source node [18].

One known issue with LBD ranking algorithms is that they tend to overweight more general or commonly cited relationships that occur across multiple journal articles. For this reason, degree-weighted path counts are used to essentially down-sample such relationships so that they do not overly influence the returned source node ranking. The mathematics for this process has been previously described [18]. The revised ULARA process was rigorously tested and validated in the original study presenting SemNet 2.0 [18]. Yet, some upweighting of common, highly connected nodes remains unavoidable to some extent and must be considered when visually inspecting results. Further limitations of the methodology are discussed in Section 4.4.

Finally, once SemNet results are generated, they must be evaluated. Because SemNet 2.0 utilizes an unsupervised approach for ranking, there is no ground truth. That is, just like in all unsupervised models, there are no positively or negatively labeled data used to train the model and to test and independently validate its results. Nonetheless, stand-in evaluation of SemNet 2.0 results can be performed in one of three ways. First, it can be performed by manual inspection of a known subset of relationships by human domain experts, as was carried out when examining historical SARS [20]. Second, highly ranked source nodes that are clinically known to be important and that reappear across multiple serial simulations can be used to set a threshold of the minimum HeteSim score to be “important”, as was done in a study for Parkinson’s Disease [23]. The third approach, which is best for exploration, is to simply examine a set number of top *k* hits across each series of simulations as a means of prioritizing top-ranked results. The top *k* hits approach was utilized for the present study, as described in Section 2.5.

### 2.2. Hub Network Analysis for Deeper, Cross-Domain Text Mining

**Figure 1:**
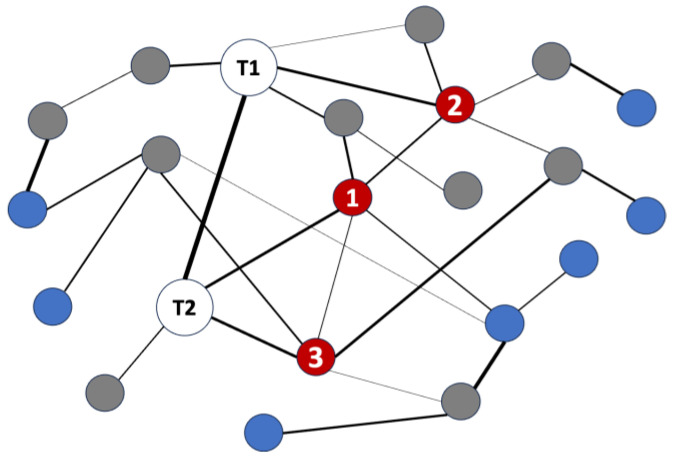
Explanation of how hub network analysis extends cross-domain text mining in SemNet 2.0. T1 and T2 represent two user-specified target nodes. Due to the computational complexity, the typical SemNet 2.0 knowledge graph search depth is n = 2 from the target node(s). The grey nodes represent more sparsely connected source nodes returned at a search depth of 2 from either T1 or T2. The nodes shown in red are the hub nodes, which are nodes that have a connection to the target at n = 2, but also have a large number of total connections to other source nodes. The utilization of hub nodes as user-specified targets for subsequent analysis effectively expands the search depth in a computationally tractable manner. Finally, the connection line width visually represents the connection frequency within the literature. Note the figure illustrates a much smaller example network to simplify conceptual explanation. The real literature network has millions of nodes and relationships, which are intractable to the human eye.

With millions of combinations of nodes comprising metapaths, it is computationally intractable to specify a large search depth in the knowledge graph. The typical search depth for a single simulation is n = 2 [18,19]. For this reason, hub network analysis is performed to enable examination of more distant cross-domain source nodes that could be very relevant to the specified target node(s) [22,23]. Hub network analysis utilizes highly ranked and well-connected source nodes returned from the initial search as target nodes for a subsequent series of SemNet 2.0 simulations. Figure 1 illustrates hub network analysis using a smaller, toy network for ease of conceptual understanding. Hub network analysis effectively extends the simulation search depth in areas of the graph most likely to return highly relevant cross-domain source nodes [22,23]. Because multiple simulations are performed as part of hub network analysis, the resultant HeteSim scores are normalized. HeteSim score normalization enables comparisons of highly ranked nodes across multiple simulations that have varying numbers of source nodes and metapaths. Details on the selection of specific hub nodes is discussed in Section 2.4.

### 2.3. Analysis Architecture and Simulation Parameters

The initial hypothesis was that participants of the RAAS pathway could be key candidates of interaction with COVID-19. Notably, resistant hypertension is the main consequence of over-stimulation of the RAAS pathway [26]. Simulations were designed to reveal important biological links between COVID-19 and resistant hypertension that may better explain their clinical connection.

**Figure 2:**
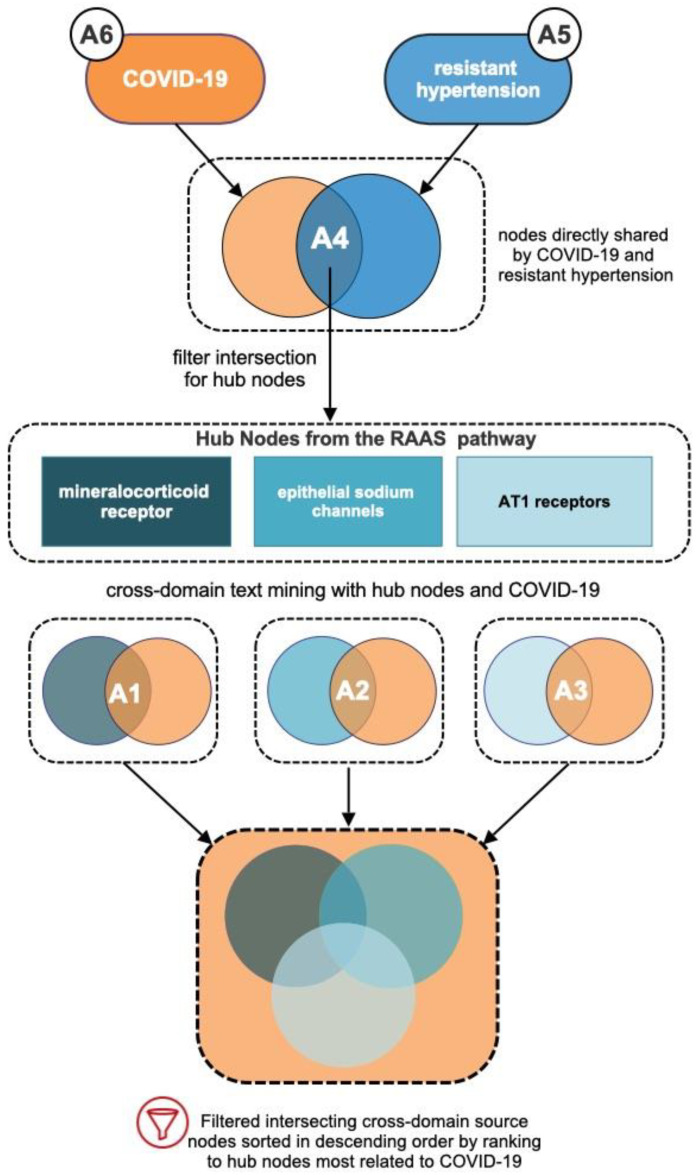
Flow diagram for the major analyses, denoted by “A”, performed to identify the biological links between COVID-19 and resistant hypertension. The analysis pipeline flows from top to bottom. Simulations were performed in SemNet 2.0. First, separate simulations were performed to identify high-ranking source nodes for resistant hypertension (A5) and COVID-19 (A6). Next, for A4, the high-ranking intersecting source nodes with prevalent metapaths were filtered to identify hub nodes. Hub nodes were selected from A4 to perform hub network analysis, which enabled cross-domain text mining of deeper relationships to COVID-19. Three hubs became target nodes for subsequent searches: mineralocorticoid receptor, epithelial sodium channels, and angiotensin type I (AT1) receptors. Separate simulations were run with each hub node (A1 for mineralocorticoid receptor, A2 for epithelial sodium channels, and A3 for AT1 receptors) to identify relationships between each hub and COVID-19. Finally, the intersection of A1, A2, and A3 was filtered and sorted in descending order by the source node’s ranking to hub nodes most related to COVID-19.

Figure 2 illustrates the flow diagram for the analyses performed. First, simulations surrounding COVID-19 (A6) and resistant hypertension (A5) were performed to reveal initial relevant source nodes. Second, the intersection of simulations A5 and A6 were utilized to get a list of high-ranking nodes related to both COVID-19 and resistant hypertension. A4 consisted of the analytical identification of three high-ranking “hub nodes” by filtering the A5–A6 intersecting source nodes by their normalized HeteSim score and metapath prevalence. A4 was used to select three hub nodes that are part of the RAAS pathway and have relationships to COVID-19: mineralocorticoid receptor epithelial sodium channels; AT1 receptor. Finally, separate simulations were performed to examine biological links between COVID-19 and each hub node: A1 examined relationships between COVID-19 and mineralocorticoid receptor; A2 examined relationships between COVID-19 and epithelial sodium channels; A3 examined relationships between COVID-19 and the AT1 receptor.

SemNet 2.0 utilizes the user-specified target nodes to identify relevant, related source nodes. The returned source node types were initially limited to the following UMLS node types: amino acids, peptides, and proteins (AAPP); hormones (HORM); and genes or genomes (GNGM). Proteins and hormones are typical components of a signaling pathway and are more likely to interact with a virus from a cell physiology standpoint. Genes or genomes were included to reveal any relationships pertaining to gene regulation by proteins. Other source node types, like social demographics or lab devices, likely would not reveal the etiology of COVID-19 to the desired biological specificity and were excluded. Table 1 summarizes the simulation input and output.

### 2.4. Justification for Target Node and Hub Node Selection

Given the primary goal of the work was to identify biological links between COVID-19 and resistant hypertension, “COVID-19” and “resistant hypertension” were selected for initial simulations in SemNet 2.0. Each concept had its own UMLS concept unique identifier (CUI) and became a target node for its corresponding simulation: A5 for resistant hypertension and A6 for COVID-19.

The underlying connections between COVID-19 and resistant hypertension are not well studied. As such, examining only existing literature that directly specifies a relationship between these two nodes at a search depth of n = 2 would be limiting. To provide greater context, A4 filtered the intersection of COVID-19 and resistant hypertension to obtain hubs for a new list of target nodes that allowed deeper exploration of less explicit literature relationships between COVID-19 and resistant hypertension. Each of the three selected hubs were part of the RAAS pathway. Again, RAAS is a critical blood and systematic vascular resistance regulator. It performs blood pressure regulation by modulating blood volume, sodium reabsorption, potassium secretion, water reabsorption, and vascular tone [14,27]. Each selected hub node (mineralocorticoid receptor, epithelial sodium channel, and angiotensin I receptor) was set as a target node, along with COVID-19, for analyses A1, A2, and A3, respectively.

A1 examined links between COVID-19 and the mineralocorticoid receptor. Mineralocorticoid receptors regulate blood pressure and maintain fluid homeostasis [28]. Aldosterone in the physiological ligand of the mineralocorticoid receptor [29]. Certain types of mineralocorticoid receptors also have antiandrogenic properties, and studies have implicated their potential effect on lowering the severity of SARS-CoV-2 infection [30].

A2 examined links between COVID-19 with the epithelial sodium channel. Epithelial sodium channels are responsible for absorbing sodium ions in the kidney and have similar functionality in the airway. Controlling sodium ions in the body is vital for maintaining homeostasis in blood pressure [31]. The RAAS pathway ensures the blood pressure stays within the healthy range by stimulating sodium reabsorption (i.e., activating epithelial sodium channels) [32]. Some studies have shown that the SARS-CoV-2 spike protein can decrease the epithelial sodium channel activity [15].

A3 examined COVID-19 with the angiotensin type I receptor (AT1). The AT1 receptor is a key receptor in the RAAS pathway. It promotes various intracellular signaling pathways resulting in hypertension, endothelial dysfunction, vascular remodeling, and end-organ damage [33]. Octapeptide angiotensin II acts on AT1 receptors, causing vasoconstriction, apoptosis, and proinflammatory effects, which can significantly affect COVID-19 patients [34].

### 2.5. Validation of Results and Determination of Themes

As noted in Section 2.1, there are three ways to evaluate SemNet 2.0 LBD results. In the present exploratory study, there was not an upfront clinical consensus that could be utilized to select the rankings of specific source node candidates as a threshold for what concepts are most important. Thus, we instead elected to utilize the top *k* hits approach, or more specifically, the top 10 hits. The top 10 candidates are reported for each source node type after analyses have been normalized. Further, the reported top 10 candidates for each analysis are then manually inspected by reviewing the full text literature. Utilizing the top 10 hits from the LBD simulations fulfills the objective of providing a reasonably sized list of prioritized multi-scalar biological concepts that could explain the clinical associations documented between COVID-19 and resistant hypertension.

The top 10 source nodes from each analysis provide a list of discrete biomedical concepts to explain the relationship between COVID-19 and resistant hypertension. However, it was also important to determine some coalesced themes that organized and explained how the highly ranked source nodes are collectively altering physiological function. A previously described bag-of-words text mining approach [22] was utilized to identify the top five defined physiological themes that provide structure for how the highly ranked source nodes explain the relationships between COVID-19 and resistant hypertension. Each relationship for the top-ranked concept was algorithmically evaluated and assigned to a theme using the full text article for each unique PubMed journal article (e.g., PMID). The “unknown” label was used to quantify relationships where a clear physiological function could not be determined. Finally, external human validation of subset of full text literature was utilized to deduce context from the highly ranked metapaths.

## 3. Results

First, metadata for the list of simulations is examined. Second, intersecting nodes more directly relating COVID-19 and resistant hypertension are analyzed. Third, hub nodes are selected and hub node analysis performed to identify deeper, cross-domain relationships that may better unearth lesser-studied etiological connection(s) between COVID-19 and resistant hypertension.

### 3.1. Analysis Metadata

HeteSim scores are utilized to determine source node relevance. However, the HeteSim scores will vary depending on the query, the number of related source nodes, and the specified source node types included in the analysis. As such, HeteSim scores can only be directly compared within a given simulation. Because multiple simulations are performed as part of cross-domain text mining, additional normalization is required to enable different layers of simulation to be fairly aggregated, filtered, and compared. As such, returned HeteSim results utilized for ranking relevant source nodes were normalized and percentile ranked. The number of metapaths and number of source nodes were taken into consideration for the normalization when aggregating, intersecting, or comparing simulations. The UMLS node types were analyzed separately for each analysis.

Table 2 summarizes the simulation output metadata. Note that A4 is omitted because it is not a separate simulation but, rather, a filtered analysis of the intersection of A5 and A6. Table 2 illustrates the number of source nodes identified in each simulation, the metapath count, the minimum HeteSim score, the maximum HeteSim score, and the mean HeteSim score. The source node count represents the number of discrete UMLS concepts identified. The metapath count represents the number of relationships between the identified source nodes and target node(s). The HeteSim score is a relevance metric used to rank the most important source nodes to the user-specified target node(s). SemNet 2.0 uses unsupervised machine learning rank aggregation to produce a HeteSim score between 0 and 1. Higher HeteSim scores represent more relevant or important source nodes.

### 3.2. Analysis of Intersecting Nodes Related to COVID-19 and Resistant Hypertension

HeteSim scores for returned source nodes were normalized using min–max normalization and then percentile-ranked. A Kendall Tau-b Rank Correlation was performed on the percentile rankings for A5 and A6. The analysis resulted in τ = 0.160, indicating low correlation. Results were further filtered by looking at the frequency of source nodes within the results. Composite source nodes are those that contain a base ID with additional IDs to link it to other source nodes. For example, for a protein family, the base ID would be the protein itself, while composite IDs would contain information linking it to specific genes that encode for it. Nodes were reduced down to their base IDs, and any nodes with a frequency less than one were filtered out.

Source nodes with a high percentile ranking that were jointly related to resistant hypertension and COVID-19 were defined as most relevant for the first search layer. The highly ranked returned source nodes were separated by UMLS node type and sorted in descending order first by their relevance to COVID-19 and then by resistant hypertension. Recall that for this exploratory study, a top 10 approach was utilized to prioritize the most relevant concepts using percentile rankings of the returned normalized HeteSim score(s). To ensure adequate inclusion of each UMLS node type in the prioritized results, the UMLS node types are segregated such that the top 10 source nodes for each UMLS node type are included in the results of an analysis.

**Figure 3:**
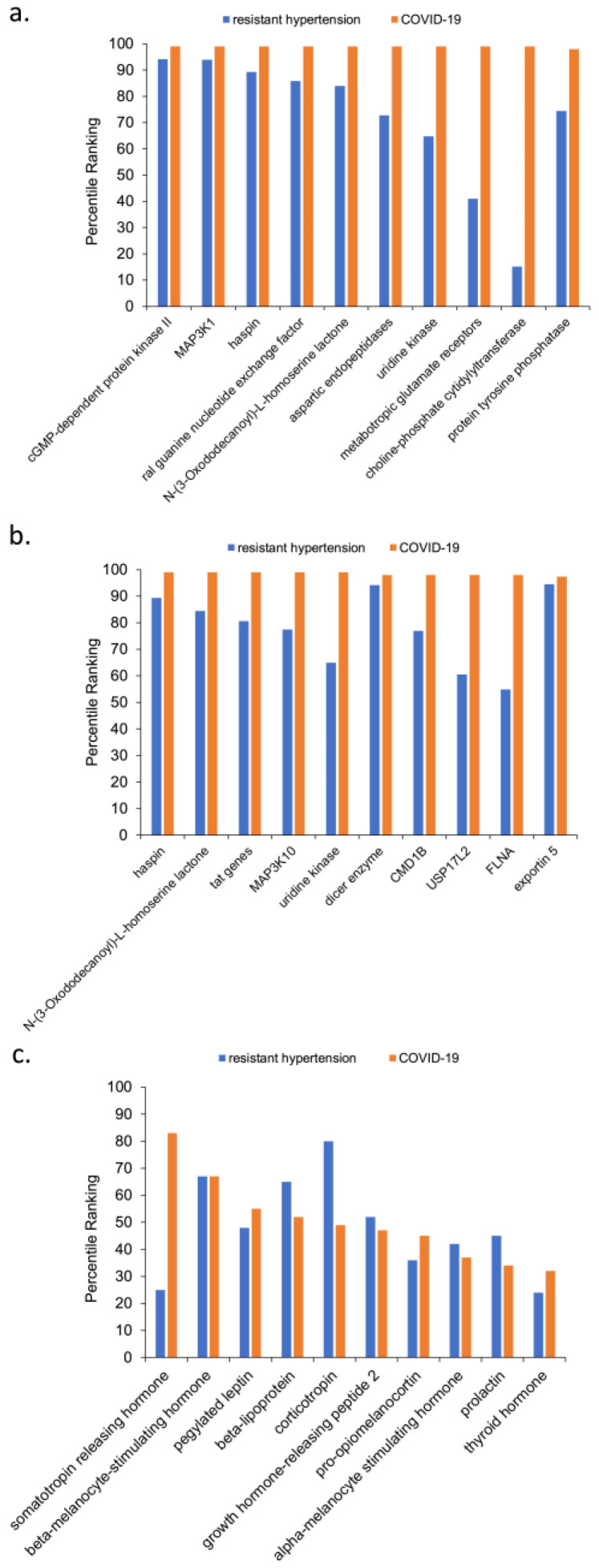
Top 10 highest scoring nodes for each UMLS node type (AAPP, GNGM, and HORM) for the intersection of analyses (A5/A6) examining source nodes related to COVID-19 and resistant hypertension. Source nodes were ranked using their normalized HeteSim scores and then percentile ranked. Source nodes are separated into the top 10 source nodes for each UMLS node type. (**a**) Top-10-ranked intersecting AAPP source nodes. (**b**) Top-10-ranked intersecting GNGN source nodes. (**c**) Top-10-ranked intersecting HORM source nodes.

The top 10 predicted source nodes of node type AAPP are shown Figure 3a, the top 10 predicted source nodes of node type GNGM are shown in Figure 3b, and the top 10 predicted source nodes of the node type HORM are shown in Figure 3c. Collectively, Figure 3 represents biomedical concepts that are well-represented in the knowledge graph as having prominent shared connections between resistant hypertension and COVID-19. The actual quantitative rankings do vary, but the scaling makes it difficult to see the difference for nodes ranking, for example, between the 98th and 100th percentiles. Because Figure 3 illustrates the predicted top 10 out of a very large list of possible source nodes, many nodes have a high percentile ranking. Notably, the large number of AAPP and GNGM source nodes in the knowledge graph contributes to their having a higher mean top 10 percentile ranking. Recall rankings are not normalized by frequency of UMLS source node type. Rather, the UMLS source node types are segregated in each analysis. Figure 3 visually illustrates why rankings must be segregated by UMLS node type to insure adequate node type inclusion. Within a given analysis, rankings should only be used to compare source nodes of the same UMLS source node type.

### 3.3. Determination of RAAS Receptor Hub Nodes Related to COVID-19

**Figure 4:**
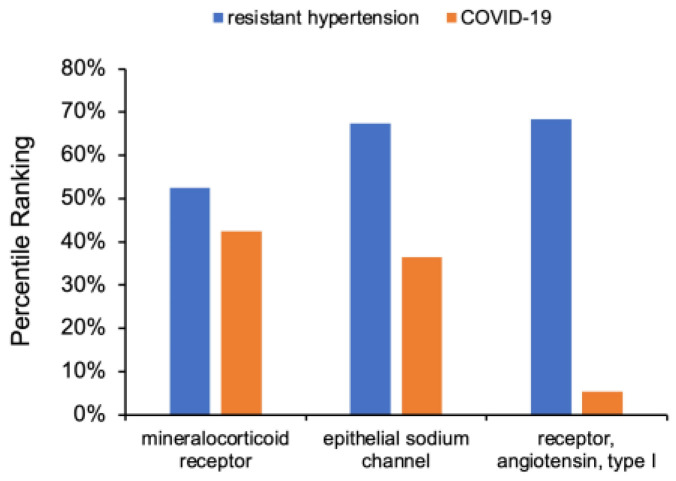
Hub node percentile ranking to COVID-19 or resistant hypertension. Mineralocorticoid receptor, epithelial sodium channel, and angiotensin type I receptor were the three selected hub nodes. Of the three hub nodes, the mineralocorticoid receptor had the strongest relationship to COVID-19. The percentile rankings are notably higher for resistant hypertension for all three hub nodes. This is due to the larger wealth of historical literature connecting the hub nodes to resistant hypertension compared to the newer concept, COVID-19, which has substantially less literature.

To perform cross-domain text mining, hub nodes must be selected. Here we selected 3 highly connected RAAS hub nodes, which also had a relatively high degree of connections to COVID-19: mineralocorticoid receptor, epithelial sodium channel, and angiotensin type I receptor. The normalized HeteSim percentile ranking for each selected hub node to either resistant hypertensin or COVID-19 is shown in Figure 4. The mineralocorticoid receptor had the highest ranking to COVID-19 (42 percentile), followed by the epithelial sodium channel (36 percentile), and angiotensin type I receptor (5 percentile). Notably, all three RAAS receptors were weighted more heavily to resistant hypertension: mineralocorticoid receptor (53 percentile), epithelial sodium channel (67 percentile), AT1 receptor (68 percentile). Having a stronger relationship to resistant hypertension compared to COVID-19 is an expected result because the connections of the RAAS receptors to COVID-19 are less established. In contrast, the literature has for many years heavily cited connections of resistant hypertension with the RAAS pathway, which strengthens those metapaths in the knowledge graph.

### 3.4. Cross-Domain Analysis with RAAS Receptor Hub Nodes and COVID-19

**Figure 5:**
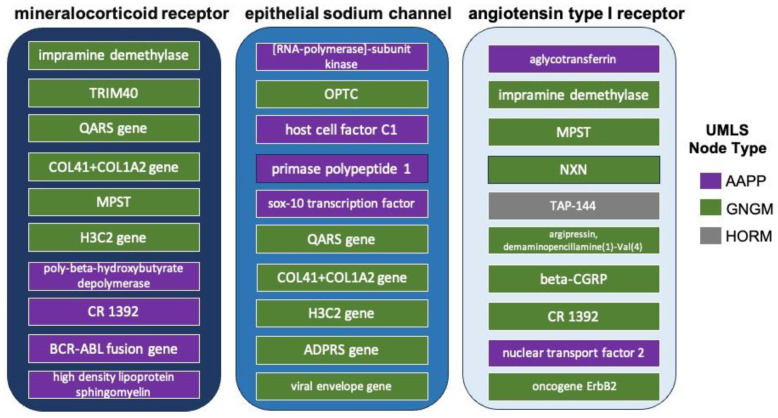
Top 10 highest scoring source nodes for each hub node simulation. The hub node and COVID-19 were specified as target nodes in SemNet 2.0 and returned source nodes were constrained to UMLS node type AAPP (purple), GNGM (green), and HORM (grey). The three hub node analyses included mineralocorticoid receptor (A1), epithelial sodium channel (A2), and angiotensin type I receptor (A3). The top 10 highest scoring source nodes were determined using normalized percentile rankings of HeteSim scores for A1, A2, and A3. Due to the extremely large number of nodes in the literature knowledge graph, the top 10 source nodes for each hub node simulation all had a percentile ranking between the 98th and 100th percentile.

The A5 andA6 simulations were an important starting point towards elucidating the etiological links between COVID-19 and resistant hypertension. However, to elucidate deeper connections, hub analysis was necessary. Figure 5 visualizes the results of the overall highest ranked source nodes for each hub node. UMLS node types AAPP (shown in purple in Figure 5) and GNGM (shown in green in Figure 5) were more prevalent in the overall rankings than HORM (shown in grey in Figure 5). The greater frequency of AAPP and GNGM node types is due to their being over-represented in the literature compared to HORM. The top 10 related source nodes to each of the hub nodes ranges between the 98th and 100th percentile.

Next, the overall highest ranked source nodes from the intersection (A4) of the three key RAAS receptors (A1-A2-A3) were used to identify new cross-domain relationships to source nodes that would not appear in the shallow initial simulations that specifically examined the intersection of resistant hypertension and COVID-19 (e.g., A5–A6). However, a drawback of keeping source nodes that were highest ranked across all three hub nodes is the over-representation of more general nodes like “enzyme structure”. Therefore, a sorting approach was utilized to account for each hub node’s previously quantified direct connections to COVID-19 shown in Figure 4. Thus, intersecting source nodes are sorted in descending order first by their percentile ranking for the mineralocorticoid receptor (A1), then by their percentile ranking for the epithelial sodium channel (A2), and finally by their percentile ranking for the AT1 receptor (A3).

**Figure 6:**
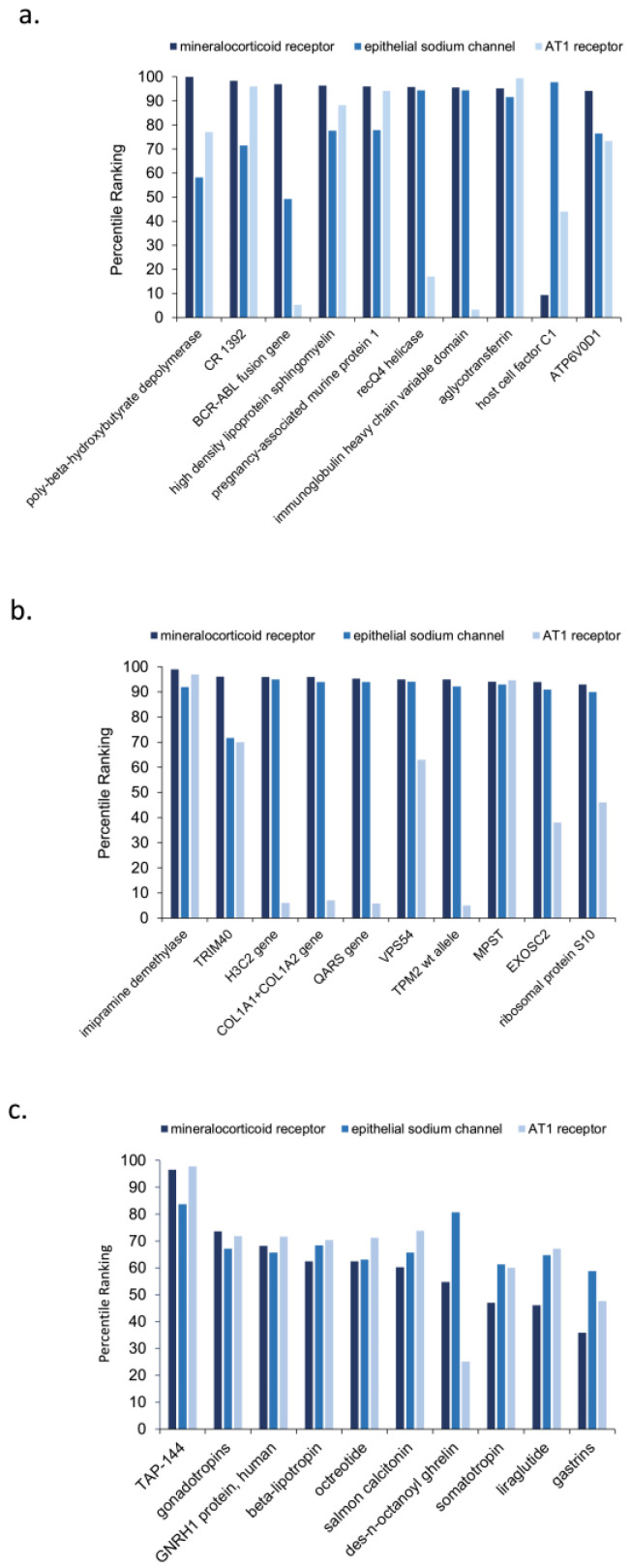
Top-10-ranked intersecting source nodes of each UMLS node type (AAPP, GNGM, and HORM) for the three hub nodes and COVID-19 sorted in descending order by rankings of A1 (mineralocorticoid receptor), then A2 (epithelial sodium channel), then A3 (angiotensin I receptor): (**a**) top-10-ranked intersecting and sorted source nodes of node type AAPP; (**b**) top-10-ranked intersecting and sorted source nodes of node type GNGM; (**c**) top-10-ranked intersecting and sorted source nodes of node type HORM. Recall the mineralocorticoid receptor was the most relevant RAAS hub node related to resistant hypertension. As such, this figure highlights nodes identified by cross-domain text mining that are predicted to be the most relevant for elucidating multi-scalar and multi-factorial links between COVID-19 and resistant hypertension.

Figure 6 illustrates the sorted cross-domain source nodes with their percentile ranking based on normalized HeteSim score separated by UMLS node type. The top-10-ranked intersecting and sorted cross-domain source nodes of node type AAPP are shown in Figure 6a, type GNGM are shown in Figure 6b, and type HORM are shown in Figure 6c. These intersecting and sorted cross-domain source nodes for each UMLS node type represent more specific, newer, or less researched relationships that may provide more nuanced context to explain the etiology between COVID-19 and resistant hypertension. Notably, the percentile rankings are inevitably overall lower in the hub node simulations (i.e., A1, A2, A3) compared to the original direct target simulations (i.e., A5, A6). For this reason, when examining cross-domain hub node analyses, the individual discrete percentile rankings of the top 10 source nodes for each node type is less informative. However, comparing the relative percentile rankings across the three hub nodes is helpful for hypothesizing potential underlying mechanisms.

### 3.5. Synthesized Biological Themes Comprised by High-Ranking Concepts

**Figure 7:**
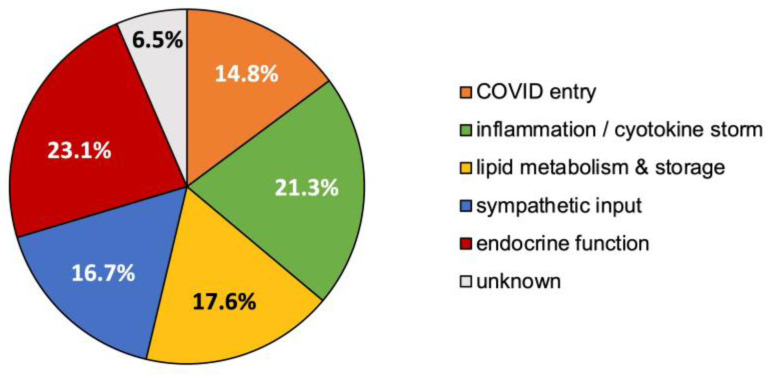
Physiological theme analysis for the top predicted biomedical concepts from all layers of analysis. The top predicted concepts primarily mapped to endocrine function, inflammation/cytokine storm, lipid metabolism and storage, sympathetic input to blood pressure regulation, or altered COVID-19 viral entry. “Unknown” was a label used to quantify concept relationships that did not clearly map to a known physiological function theme.

Examination of the high-ranking source nodes from all pertinent analyses was performed to identify a few coalesced physiological function themes. “Themes” provide clues for how the multi-scalar etiology between COVID-19 and resistant hypertension may fit together. These themes were initially produced by quantitative analysis of physiological tiers in the network with a simple bag-of-words text mining approach that has been previously described [22]. Each of the predicted top 10 concepts for each layer of analysis was included in the theme analysis. Relationships to the concepts were counted to determine their association frequency. The number of times a concept was attributed to a theme for each unique PMID (e.g., for each full-text article with a unique PubMed identifier) was counted and expressed as a percentage of the total as shown in Figure 7. In short, each reported percentage corresponds to the percentage of overall relationships attributed to the theme. Themes were validated using manual human visual inspection of a subset of full-text articles comprising the identified high-ranking metapaths.

As shown in Figure 7, the most frequent associated physiological functions for the top predicted concepts (e.g., source nodes) fell under one or more of the following themes:

- Concept relationships that modulate endocrine function, predominantly via the hypothalamic–pituitary–adrenal axis, 23.1%;
- Concept relationships mapping to inflammation and/or the cytokine storm, 21.3%;
- Concept relationships associated with lipid storage, metabolism, or atherosclerosis, 17.6%;
- Concept relationships that alter sympathetic drive for blood pressure regulation, 16.7%;
- Concept relationships that explained the entry or altered uptake of COVID-19, 14.8%;
- Concept relationships that did not clearly map to one of the above specified themes were labeled as “unknown”, 6.5%.

## 4. Discussion

LBD simulations were performed in SemNet 2.0 to identify connections between COVID-19 and resistant hypertension. In this section, interpretation and context is provided for the top-ranked source nodes. First, the more shallow or direct connections are discussed, which included returned source nodes that directly intersect with COVID-19 and resistant hypertension. Second, the deeper, cross-domain relationships revealed are discussed, which may better explain pertinent underlying etiology that connects COVID-19 and resistant hypertension. Third, the major biological themes of returned source nodes from both direct and cross-domain simulation are synthesized. Synthesized themes provide an overarching view of predicted shared etiology between COVID-19 and resistant hypertension. Finally, LBD and study-specific limitations are presented.

### 4.1. Explicit Literature Relationships between Resistant Hypertension and COVID-19

The intersection of the A5-A6 simulations examined direct connections shared by resistant hypertension (A5) and COVID-19 (A6) segregated by UMLS node type (AAPP, GNGM, and HORM). Below each high-ranking source node is discussed with cited context in the literature.

The top-ranking AAPP node types for the intersection of COVID-19 and resistant hypertension are discussed below:

- *haspin*—Haspin is a mitotic kinase for Histone H3, is regarded as a promising anti-tumor therapy; it is overexpressed in malignant tissues due to its requirement for cancer cell proliferation [35].
- *ral guanine nucleotide exchange factor*—Ral guanine nucleotide exchange factor was previously identified as an important signaling component that regulates transcriptional responses in myocardial cells [36]. It also promotes cardiomyocyte survival and inhibits cardiac fibrosis [37]. Interestingly, the Ral and Ras pathways have also been implicated in myeloid differentiation [38] and especially the BCR-ABL mutation that causes chronic myeloid leukemia [39].
- *N-(3-Oxododecanoyl)-L-homoserine lactone*—Prior work found that N-3-oxododecanoyl homoserine lactone exacerbates endothelial cell death by inducing receptor-interacting protein kinase 1-dependent apoptosis [40]. It also been reported to induce apoptosis in various types of tumor cells, primarily through a mitochondrial pathway [41]. Notably, N-(3-Oxododecanoyl)-L-homoserine lactone is categorized as a bitter taste receptor. Neutrophils, monocytes, and lymphocytes can express bitter taste receptors being involved in immune response [42]. For this reason, N-(3-Oxododecanoyl)-L-homoserine lactone was hypothesized as one possible therapy for COVID-19 [43].
- *uridine kinase*—The UK2 gene encodes a uridine kinase protein that catalyzes the phosphorylation of uridine and cytidine to uridine monophosphate (UMP) and cytidine monophosphate (CMP). Uridine is phosphorylated to nucleotides [44]. Most notably, the presence of uridine in RNA and DNA nucleotides allows the SARS-CoV-2 enzyme to cleave RNA because SARS-CoV-2 is a uridine-specific endoribonuclease. Its active site binds to the uridines that are phosphorylated to RNA and DNA nucleotides, allowing the enzyme to cleave RNA [45]. Uridine nucleotides also play an important role in achieving homeostasis in the vascular system. Augmented contractile responses to uridine nucleotides in the femoral arteries of spontaneously hypertensive rates were much higher than in non-hypertensive rats [46]. Uridine kinase has long been known to be involved in the heart physiology. Earlier studies also showed a positive correlation between thyroid hormone and presence of uridine kinase in cardiac cells [47]. More recent work showed that uridine has a hypoglycemic effect that protects against diabetes-mediated functional and structural damage to cardiac mitochondria and disruption of mitochondrial quality-control systems in the diabetic heart [48].
- *metabotropic glutamate receptors*—Chronic stimulation of group II metabotropic glutamate receptors in the medulla oblongata attenuates hypertension development in spontaneously hypertensive rats [49]. Group III metabotropic glutamate receptors regulate hypothalamic pre-sympathetic neurons through opposing presynaptic and postsynaptic actions in hypertension [50]. Metabotropic glutamate receptors are also important in the regulation of steroidogenesis in the human adrenal gland [51]. As for ties to COVID-19, SARS-CoV-2 uses metabotropic glutamate receptor subtype 2 as an internalization factor to infect cells [52].
- *aspartic endopeptidases*—This group of endopeptidases is closely tied to the RAAS pathway that induces hypertension and has also been utilized in HIV therapies [53]. Aspartic endopeptidases have also been investigated to reduce dexamethasone-induced hypertension and associated fibrosis in rat models [54]. Their role in the RAAS pathway has also been explicitly noted during and after COVID-19 infection [13].
- *MAP3K1*—MAP3K1 stands for MAP kinase kinase kinase 1. MAP kinase signaling plays a prominent role in the RAAS pathway. Prior work has shown that angiotensin II up-regulates angiotensin I-converting enzyme (ACE), but down-regulates ACE2 via the AT1-ERK/p38 MAP kinase pathway [55]. Anti-cytokine therapies targeting JAK-STAT, such as Ruxolitinib, were shown to have a lesser incidence of cardiovascular adverse events compared to steroids given for COVID-19 [56].
- *protein tyrosine phosphatase*—One type of protein tyrosine phosphatase, CD45, was found to be altered in the leukocytes of COVID-19 patients [57]. Protein tyrosine phosphatase has been cited as having a role in both essential hypertension [58] and pulmonary hypertension [59]. Not surprisingly, tyrosine kinase inhibitors for CML therapy also cause similar adverse events [22]. Similarly, the related JAK pathway has overlap with both COVID-19 and CML [60]. Imatinib, a first-line therapy for CML, was tried as a potential COVID-19 therapy based on two hypothesized roles [61]: (1) potential intralysosomal entrapment of imatinib may increase endosomal pH and effectively decrease SARS-CoV-2/cell fusion, (2) the kinase inhibitory activity of imatinib may interfere with budding/release or replication of SARS-CoV-2.
- *cGMP-dependent protein kinase II*—Angiotensin II/AT2 receptor-induced vasodilation in stroke-prone spontaneously hypertensive rats involves nitric oxide and cGMP-dependent protein kinase [62] and also has shown to impact hypertrophy and fibrosis [63].
- *choline phosphate cytidylyltransferase*—Choline phosphate cytidylyltransferase responsible for regulating phosphatidylcholine content in membranes. Fetal lung fatty acid synthase and choline phosphate cytidylyltransferase activities are increased by glucocorticoids [64].

The top-ranking GNGM node types for the intersection of COVID-19 and resistant hypertension are discussed below:

- *MAP3K10*—MAP3K10 stands for MAP kinase kinase kinase 10. MAP3K10 activity has been cited in many cancers, namely pancreatic cancer. However, it has also been implicated in the atherosclerotic inflammatory process [65].
- *tat genes*—The tat (transactivator of transcription) gene transcribes the tat protein, a required transactivator for expression of full-length viral genes, which influences expression of cellular inflammatory genes [66]. Tat has also been implicated in pathways that to modulate angiotensin II-induced medial hypertrophy [67].
- *exportin 5*—Exportin 5, also known as XPO5, is a gene that has been implicated in pregnancy-induced hypertension [68]. While XP05 was thought to modify viral expression, there was no difference in XPO5 expression in COVID-19-infected versus control patients [68].
- *CMD1B*—CMD1B is associated with pregnancy-induced hypertension and familial dilated cardiomyopathy [69]. One case report in a young COVID-19 patient with Emery–Dreifus muscular dystrophy and a family history of dilated cardiomyopathy found indication of viral myocarditis [5].
- *FLNA*—FLNA defects can be lethal as it leads to skeletal defects and defects which cause severe cardiac malformations [70].
- *USP17L2*—Ubiquitin-Specific Peptidase 17-Like Family Member 2 is most known for its role in cancers [71]. USP17 substrates populate two pathways that drive cell cycle progression: one that promotes and one that inhibits. This dual path could explain its both pro-cancer and anti-tumor effects.
- *dicer enzyme*—Dicer enzyme is a microRNA that has been associated with pregnancy-induced hypertension [68] as well as the brain renin–angiotensin II-induced hypertension and cardiac hypertrophy [72]. More recently, one isoform of Dicer, named antiviral Dicer (aviD), was found to protect tissue stem cells from RNA viruses—including Zika virus and severe acute respiratory syndrome coronavirus 2 (SARS-CoV-2)—by dicing viral double-stranded RNA to orchestrate antiviral RNAi [73].

The top-ranking HORM node types for the intersection of COVID-19 and resistant hypertension are discussed below:

- *somatotropin-releasing hormone*—Somatropin is responsible for modulating the release of growth hormone. There is a known delicate balance between endocrine and autonomic function that impact blood pressure. Somatropin hormone, thyrotropin, gonadotropin-releasing hormone, and corticotropin-releasing hormone and others impact the RAAS pathway via angiotensin II to modulate blood pressure [74]. Individuals with isolated congenital GH deficiency due to a GHRH receptor gene mutation appear to cope better with SARS-CoV-2 infection than controls [75]. Likewise, somatotropin-releasing hormone has been shown to be highly correlated to COVID-19. Observations show growth-stimulating hormone may pose as a predictor for severity of post-COVID-19 symptoms while also appearing somewhat relevant to resistant hypertension [76]. Prior research illustrated the anti-inflammatory properties of growth-stimulating hormone receptor antagonists to resistant hypertension [77].
- *melanocyte-stimulating hormone*—Melanocyte-stimulating hormones (MSHs) are involved in energy metabolism and in inflammation. While melanocyte-stimulating hormones are prevalently acknowledged to have anti-inflammatory and anti-hypertensive properties, their impact and role as a potential adjuvant treatment for COVID-19 remains under scrutiny [78]. Additionally, alpha- and gamma-MSH acutely elevate blood pressure and heart rate through central stimulation of sympathetic nervous outflow [79]. One study implicated neuropeptide Y and alpha-melanocyte-stimulating hormone in hypothalamic regulation of sympathetic nervous system activity [80].
- *pegylated leptin*—Pegylated leptin has been shown to play an important role in development via hypothalamic trophic factors [81]. Leptin also has prominent roles in energy metabolism. Leptin resistance causes obesity [82].
- *beta-lipotropin*—Beta-lipotropin was initially reported to stimulate melanocytes to produce melanin. Later, it was found to perform lipid-mobilizing functions such as lipolysis and steroidogenesis [83].
- *corticotropin*—Also known as adrenocorticotropin hormone, corticotropin is produced by the anterior pituitary gland and is an important part of the hypothalamic–pituitary–adrenal axis. As such, it has direct roles in the stress response that increases blood pressure. Autopsy studies on patients who died from the SARS outbreak in 2003 had shown degeneration and necrosis of the adrenal cortical cells [84]. Additionally, SARS-CoV-2 (e.g., COVID-19) was identified in the adrenal glands, hinting towards a direct cytopathic effect of the virus and altered cortisol dynamics [84].
- *growth-hormone-releasing peptide-2*—Also known as GHRP2, it is a synthetic agonist of ghrelin, a gut peptide that binds to the growth hormone secretagogue receptor. Ghrelin increases growth hormone secretion and appetite initiation [85].
- *pro-opiomineralocorticoid*—This hormone is synthesized in the anterior pituitary and is part of the central mineralocorticoid system. It has been mentioned as part of the hypothalamic–pituitary autoimmunity seen in COVID-19 patients [86]. Mineralocorticoid receptors are thought to relieve the endothelial and systemic inflammatory mechanisms of respiratory viruses [87].
- *prolactin*—Prolactin is best known for its role in enabling milk production. However, it also has a role in immunity. For this reason, it was hypothesized that controlled augmentation of prolactin could provide protective benefits for patients infected with COVID-19 [88]. Anecdotal evidence was presented in seven patients when prolactin was initially recommended as a possible repurposed therapy [88].
- *thyroid hormones*—Thyroid hormones were a recurring top-ranked source node. The thyroid gland also express angiotensin-converting enzyme 2 (ACE2), the main protein that functions as a receptor to which SARS-CoV-2 binds to enter host cells. Immune system cells are targets for thyroid hormones and T3 and T4 modulate specific immune responses, including cell-mediated immunity, natural killer cell activity, the antiviral action of interferon and proliferation of T- and B-lymphocytes [89]. Thyroid pathology is a known event during and immediately after COVID-19 infection. There has been numerous established cases of COVID-19 induced pathology [89] ranging from thyrotoxicosis to suppressed thyroid function, which are largely attributed to the “cytokine storm” [90]. However, one study found that the thyroid hormones T3 and T4 are decreased during active COVID-19 infection compared to baseline [91].

### 4.2. Intersection of RAAS Hub Nodes and Relationship to COVID-19 and Resistant Hypertension

Hub node analysis allowed a deeper cross-domain probing of the knowledge graph to find less represented relationships that have more distant literature connections to COVID-19 and/or resistant hypertension. The returned intersecting and filtered A1-A2-A3 source nodes (e.g., mineralocorticoid receptor, epithelial sodium channel, AT1 receptor) from cross-domain hub analysis shown in Figure 6 are more likely to convey nuanced information. As such, these predictions may help prioritize future experiments to further elucidate their relationship(s) between COVID-19 and resistant hypertension.

The top-ranking AAPP node types for the intersection of the three RAAS hub nodes and COVID-19 are discussed in context with the supporting literature below:

- *poly-beta-hydroxybutyrate depolymerase*—It is most known for its role in bacterial microbe metabolism. It is produced by penicillium expansum. Notably, the enzyme requires essential disulphide bonds (cystine residues) and tyrosine to maintain the native enzyme structure [92]. Moreover, patients with severe COVID-19 were found to have elevated levels of branched-chain amino acids and beta-hydroxybutyrate [93].
- *CR 1392*—It is most known for its role on pancreatic exocrine function and insulin release [94,95]. Glucose dysregulation among patients the COVID-19 is well known [96]. The inflammation of COVID-19 is believed to contribute to increased blood glucose levels. COVID-19 patients with new or worsened glucose dysregulation requiring additional or new insulin administration were associated with poorer outcomes in a study of 456 patients [96].
- *BCR-ABL fusion gene*—Within the list of highest percentile-ranked amino acids and proteins in Figure 6a, the BCR-ABL fusion gene possesses a percentile ranking for mineralocorticoid receptors that is nearly 50% higher than the next greatest percentile ranking for the source nodes of interest. While most clinicians associate the presence of BCR-ABL fusion gene with chronic myeloid leukemia (CML), about 10% of healthy control patients express at least some measurable copies in their peripheral blood [97]. Prior work relating COVID-19 to CML concisely summarizes the role of the gene in upregulating fusion transcripts and proteins, ultimately intensifying the presence and activity of tyrosine kinase within the body [98]. Recent extensive investigation of tyrosine kinase has revealed its direct association in vasculopathy as well as its antihypertensive properties from its interactions with endothelial receptors [99]. Such findings could explain the well-predicted increase in cardiovascular adverse events, including hypertension, in CML patients on tyrosine kinase inhibitor therapies [22]. Since protein kinase in integral in the signaling process of mineralocorticoid receptors both on and within the nucleus of endothelial cells [100], it can be hypothesized that COVID-19 provokes resistant hypertensive tendencies partially by manipulating the expression of BCR-ABL in the mineralocorticoid receptor pathway, decreasing the activity of tyrosine kinase [100].
- *high-density lipoprotein sphingomyelin*—It is part of high-density lipoprotein (HDL). HDL levels are an inverse risk factor for cardiovascular diseases, and sphingomyelin is the second most abundant phospholipid component and the major sphingolipid in HDL [101]. In one large-scale study, patients with severe COVID-19 were reported to have low levels of total cholesterol, HDL-cholesterol, and LDL-cholesterol, but elevated levels of triglycerides [93].
- *immunoglobulin heavy chain variable domain*—It is important for binding the antigen and the chain variable constant domains necessary for successful B cell maturation. The heavy chain variables have been suggested as a point of entry for useful applications for prophylaxis and therapy of COVID-19 alone or in combination [102].
- *pregnancy-associated murine protein 1*—This particular protein is from the mouse domain. However, there could be some relevant ties to clinical COVID-19. Prior work demonstrated a distinct difference in immune modulation between the non-pregnant and pregnant states in COVID-19 patients, which may provide some insight into the pathogenesis of COVID-19 and perhaps explain the more severe outcomes observed in pregnant women [103].
- *recQ4 helicase*—RecQ4 helicase is a family of helicase enzymes that has been shown to be important in genome maintenance and stability [104]; they catalyze the reaction of ATP and water to drive the unwinding of paired DNA. Relatively little is known about recQ4 other than its important role in genetic stability. Other helicases besides recQ4 have been suggested as potential therapies for COVID-19 based on their antiviral activity [105].
- *HCF1*—HCF1 is involved in control of the cell cycle and has regulatory roles in a multitude of processes related to transcription. Transcriptional coactivator HCF-1 couples the histone chaperone Asf1b to HSV-1 DNA replication components [106]. Recent works point to HCF1 as being a putative longevity determinant [107]. Additionally, prior work with herpes simplex virus (HSV) illustrated that multiple chromatin modulation components associated with HCF-1 lead to the initiation of immediate early gene expression in HSV [108].
- *ATP6V0D1*—ATP6V0D1 encodes a component of vacuolar ATPase (V-ATPase) and is involved in lysosome homeostasis. Clinically, a high expression of ATP6V0D1 was correlated with prolonged survival of patients with pancreatic ductal adenocarcinoma [109]. It has been shown to correlate with thyroid hormones [110] and proinsulin processing [111]. Collectively, these data suggested that the S protein from COVID-19 increased V-ATPase in SARS-CoV-2 infection, which provided a microenvironment easier for the cleavage of S protein by making the activation of inflammatory cells in the respiratory epithelium easier [112].

The top-ranking GNGM node types for the intersection of the three RAAS hub nodes and COVID-19 are discussed in context with supporting literature below:

- *imipramine demethylase*—Imipramine is a tricyclic antidepressant that undergoes demethylation as part of metabolism. Imipramine increases dopamine in the striatum [113]. Imipramine demethylation is associated with the CYP2D6 and CYP2C19 genotype. While there is no straightforward reason for its predicted association with COVID-19, it is known that dopamine increases hypertension [114]. A meta-analysis examining the association of anti-depressants with COVID-19 incidence and severity found that most either had no impact or had have slight protective effects [115].
- *TRIM40*—Tripartite motif (TRIM)-containing proteins are E3 ubiquitin ligases that possess crucial regulatory functions in innate immunity [116]. In particular, they attenuate antiviral immune response [117]. TRIM40 also has associations with IgA nephropathology, which causes kidney disease; it is thought to suppress IgA1-induced GMC proliferation by inhibiting the activation of NLRP3 inflammasome [116]. TRIM40′s associations with kidney disease and regulatory functions in immunity both align with a role in resistant hypertension and COVID-19, respectively.
- *QARS gene*—Encoding glutaminyl-tRNA synthetase QARS has been implicated in progressive microcephaly, severe seizures in infancy, atrophy of the cerebral cortex and cerebellar vermis, and mild atrophy of the cerebellar hemispheres [118]. Its repeated selection by the algorithm as a potential important source node for the relationship between COVID-19 and resistant hypertension remains unclear.
- *COL1A1+COL1A2 gene*—are collagen I and II genes that encode primarily for connective tissue. COL1A1+COL1A2 gene mutations are most implicated in osteogenesis impefecta (OI), which is also known as brittle bone disease. Additionally, patients with COL1A1+COL1A2 gene mutations have shown various forms of cardiovascular pathology, particularly regurgitation of the heart valves, attributed to poor collagen [119]. Interestingly, a small study examining pediatric OI patients found that they had no difference in outcome after COVID-19 infection [120]. Moreover, two therapies used for OI, mesenchymal stem cells and decidua stromal cells were used to treat SARS-CoV-2 coronavirus-induced disease [121] as an anti-inflammatory treatment to reduce COVID-induced cytokine storm.
- *MPST*—The biological function of MPST remains unclear. It may be involved in cyanide detoxification, biosynthesis of thiosulfate, production of the signaling molecule hydrogen sulfide, or the degradation of cysteine. Sulfur metabolism in the liver has been strongly correlated with hypertension in animal models [122]. Sulfur dioxide also increases pulmonary hypertension [123]. Interesting, air pollutants like sulfur dioxide were found to be associated with increased incidence of COVID-19 [124]. Additionally, liver dysfunction marked by elevation of lipoproteins X and Z has been found in patients with severe COVID-19 [93].
- *H3C2 gene*—It encodes for histone 3, which is fundamental in development. H3C2 has been associated with several pediatric gliomas [125]. Notably, haspin, which encodes for histone, was found to be among the top-ranking nodes for both AAPP and GNGM node types in analysis A4, which examined the intersecting source nodes for resistant hypertension and COVID-19.
- *VPS54*—VPS54 is most known for its role in motor pathology, such as ALS phenotypes, including the wobbler mouse [126]. However, the vacuole protein sorting gene VPS54 was also recently shown to be required for extracellular virus (EV) formation in monkeypox infection [127]. It is possible VPS54 may play a similar role in COVID-19.
- *TPM2*—TPM2, which encodes beta-tropomyosin, is well known for its role in atherosclerosis [128]. Downregulation is also associated with various cardiomyopathies and coronary artery disease [129]. Coronary artery disease is a known risk factor for poor COVID-19 outcomes [130].
- *EXOSC2*—Low expression of EXOSC2 was found to protect against clinical COVID-19 and impedes SARS-CoV-2 replication [131]. Likewise, aggregating COVID-19 GWAS statistics revealed an association between increased expression of EXOSC2 and higher risk of clinical COVID-19 [131]. EXOSC2 is a component of the RNA exosome that LC-MS/MS analysis demonstrated an interaction between the SARS-CoV-2 RNA polymerase and the majority of human RNA exosome components [131]. Additionally, EXOSC2 has been linked to sudden cardiac death [132] due to cardiac conduction abnormalities and arrhythmia from QTc prolongation. As such, EXOSC2 has clear ties to both COVID-19 and cardiovascular disease.
- *ribosomal protein S10*—The role of ribosomal protein S10 is not clear with COVID-19. However, there is an association with lupus autoantibodies [133]. COVID-19 and subsequent cytokine storm was associated with both new onset lupus [134] as well as lupus flares in existing patients with systemic lupus erythematosus (SLE) [135]. Additionally, hydroxychloroquine, a common drug for lupus, was found in computer simulations to illustrate a potential benefit [20]. While hydroxychloroquine was initially tried as an adjuvant therapy for COVID-19, its efficacy for COVID-19 was ultimately found to be controversial [135]. Hydroxychloroquine for COVID-19 was largely stopped over concerns of sudden cardiac death from arrhythmia [136]. Interesting, an increase in new onset SLE has been noted after COVDI-19 infection [134].

The top-ranking HORM node types for the intersection of the three RAAS hub nodes and COVID-19 are discussed in context with supporting literature below:

- *TAP-144*—TAP-144 is also known as leuprorelin or leuprolide, which is a gonadotropin-releasing hormone analogue family of medications. Side effects may include high blood sugar and problems with the pituitary gland. Interestingly, previous artificial intelligence work identified leuprolide as a possible repurposed drug for COVID-19 based on its structural biology [137]. TAP-144 has a high binding affinity to COVID-19 and was thought to modify the functional ability of the spike protein. Leuprolide was validated by molecular docking against the spike protein complex with ACE receptor [137].
- *des-n-octanoyl ghrelin*—Ghrelin is involved in appetite stimulation and growth hormone release. Des-n-octanoyl ghrelin has some distinct functions from ghrelin. Namely, the lack of acylation prevents binding to the ghrelin receptor and growth hormone release. Interestingly, patients with cardiovascular events were found to have lower levels of des-acyl ghrelin at baseline than those without cardiovascular events [138].
- *beta-lipotropin*—Both beta-lipotropin and beta-endorphin are present in cardiac tissue. The amounts and ratio of beta-endorphin and beta-lipotropin in the heart appear to be modulated by testosterone propionate [139]. Circulating beta-endorphin and beta-lipotropin concentrations increase after the administration of acetylcholine or serotonin agonist drugs [140]. Patients with heart disease, namely congestive heart failure, were found to have lower amounts of beta-lipotropin than control patients [141].
- *gonadotropin*—Gonadotropins and GNRH1 protein have been most cited based on suspected changes in fertility after COVID-19 infection [142]. Sub-clinical hypogonadism has been seen following COVID-19 infection in males characterized by increased LH and decreased testosterone production [143].
- *human salmon calcitonin*—has been recognized in thyroid extracts of normal subjects and of patients with medullary carcinoma [144]. Multiple studies have investigated the impact of human salmon calcitonin on cancer risk [145].
- *liraglutide*—Liraglutide is anti-diabetic medication utilized to treat hyperglycemia due to type 2 diabetes. New onset diabetes is another disease found to be a sequala of COVID-19 [10,11]. Higher blood sugar tends to happen during and after COVID-19 infection [96,146] and is a comorbidity with other reported endocrine dysfunction brough on or exacerbated by COVID-19 [91].
- *octreotide*—Octreotide has been successfully utilized to treat portal hypertension in the liver associated with cirrhosis or other liver pathology [147]. As noted earlier, markers of hepatic dysfunction are also prominent in severe COVID-19 illness [93]. One study reported a patient with acromegaly and pre-diabetes with severe respiratory distress from COVID-19 that responded quite positively to the administration of octreotide to improve COVID-19 outcome [148]. Additionally, another structural biology simulation predicted octreotide as a viable repurposed drug for COVID-19 [149]. Acromegaly has been hypothesized by other studies to be a point of emphasis in COVID-19 etiology and treatment [150].
- *somatotropin*—is a recombinant form of human growth hormone. In a clinical study of 456 patients, growth hormone and IGF-1 deficiency were found in COVID-19 cases with lung involvement, regardless of age or gender; COVID-19 infection progressed worse in GH and IGF-1 deficiency [151]. Another study examining COVID-19 inflammation in cellular model found that growth hormone and estradiol improved inflammation, but testosterone had the opposite effect [152]. However, a potential complicating factor of growth hormone treatment for COVID-19 is that acromegaly, a state of endogenous GH excess, results in myocardial hypertrophy and decreased cardiac performance with increased cardiovascular mortality [153].
- *gastrins*—Hypertension is related to impaired metabolic homeostasis and can be regarded as a metabolic disorder [154]. Interestingly, an in silico modeling study examining molecular dynamics suggested that pentagastrin, a synthetic polypeptide that has effects like gastrin when given parenterally, could be a viable drug for COVID-19 [155]. Gastrin-releasing peptide has long been held for its possible role as a target for inflammatory disease [156], including cardiovascular disease, gastrointestinal disease, pulmonary disease, and of course, its roles in endocrine disorders, namely glucose metabolism.
- *thyroid hormone*—Thyroid hormone was a recurring highly ranked source node identified during both direct simulation and cross-domain analysis. It has been hypothesized that hyperinflammation, as reflected by the secretion of cytokines, might induce thyroid dysfunction among patients with COVID-19. Thyroid hormone involvement in the acute phase of symptomatic COVID-19 and its possible associations with cytokine levels and mortality risk have been explored [157]. Findings suggest that fluctuations of TSH levels in patients with COVID-19 may be influenced by circulating IL-8, IL-10, IL-15, IP-10, and GM-CSF as previously described in autoimmune thyroid diseases [157]. Thyroid sequala are well-documented in COVID-19 [84,89,91,157]. There are also documented correlations with thyroid hormone and salmon and human calcitonin peptides and uridine kinase [47], of which salmon calcitonin peptide was also a highly ranked AAPPs in the present study [144].

### 4.3. Explaining Synthesized Biological Themes Comprised by High-Ranking Concepts

The relationships to the high-ranked source nodes were evaluated using discrete unique full text PubMed journal articles and then classified into physiological themes. The majority of relationships were mapped to six major physiological themes: altered endocrine function, 23.1%; inflammation or cytokine storm, 21.3%; lipid metabolism, storage, atherosclerosis, 17.6%; sympathetic input to blood pressure regulation, 16.7%; altered entry of COVID-19 virus, 14.8%; and unknown, 6.5%.

**Figure 8:**
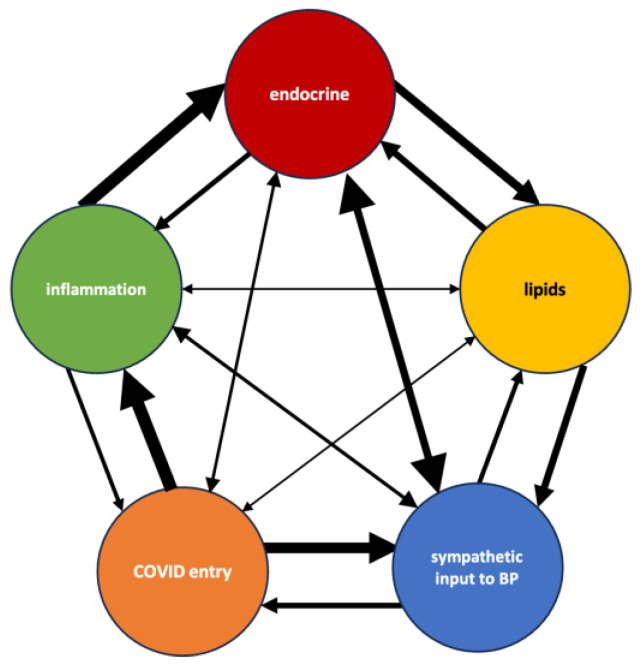
Pictorial diagram illustrating relationships between the common physiological function themes that were mapped using the connections of highly ranked source nodes. The arrow thickness approximates the relative weight of the relationship between themes.

The dominance of these themes can be an anecdotally validated via existing clinical association studies. Both resistant hypertension and COVID-19 act through the RAAS pathway. Thus, patients with existing clinical or even preclinical tendencies to have disruption in the RAAS pathway could explain why hypertension is a known risk factor for COVID-19. Likewise, the inflammation and cytokine storm that occurs in some COVID-19 patients can increase hypertension through RAAS and the hypothalamic–pituitary–adrenal axis [84]. The cytokine storm can also impact endocrine function, namely thyroid hormone [90] and growth hormone [151], both of which were recurring top predict concepts in multiple SemNet 2.0 analyses in the present study. Furthermore, endocrine dysfunction changes, such as decreased thyroid hormone then propagates to dysfunctions in lipid storage, metabolism, and risk of atherosclerosis through dyslipidemia. Recall thyroid disorders [89,91], diabetes, and dyslipidemia have also been identified in clinical populations as sequela to COVID-19 infection [11]. Finally, neurologic regulatory changes, including to the neurons of the gut, may change the ion, neurotransmitter, or gain regulation for the neurons that control sympathetic regulation of blood pressure [154]. For example, melanocyte-stimulating hormone was predicted as a top target in multiple simulations and is known to increase sympathetic input to blood pressure regulation [79]. Figure 8 illustrates the relationships between themes.

### 4.4. Study Limitations

No form of model or analysis is perfect. Likewise, LBD has known limitations. Below, we discuss technical limitation of the algorithm and applied limitations in terms of interpreting the present study’s results.

#### 4.4.1. Technical Limitations

Technical limitations include limitations of the input data, algorithm, and validation or generalizability of results. The input data to LBD are quite literally the scientific literature, which has its own imperfections. The SemNet 2.0 algorithm data input are the text from abstracts from about 33+ million journal articles contained within PubMed. SemNet 2.0 does not utilize a publication quality index, citation index, etc., to weight data sources. Rather, all data sources are treated equally. Also, SemNet 2.0 cannot account for context-related changes over time. Rather, all extracted relationships are added to the graph. The underlying text to metapath translation algorithm [158] has a 75–85% accuracy, which remains a fairly state-of-the-art accuracy for biomedical text natural language processing. In the future, improved named entity recognition and relationship extraction algorithms will further raise the translation accuracy for SemNet 2.0 and other LBD tools. Finally, the ranking is performed by a revised unsupervised learning rank aggregation [18], which means there is no known ground truth from which to test the model. However, external COVID-19 clinical studies that tested some of the repurposed drug predictions made by a LBD link prediction algorithm employing SemNet [20] found at least 40% of its predictions to be clinically successful [21]. As such, there are external data to suggest the SemNet family of LBD algorithms is useful for exploration and prioritization of relationships to targets of interest. In the present exploratory study, which examined a topic with less discrete clinical consensus, the top hits (i.e., top 10) approach was utilized. Given there are so many related source nodes, a limitation is that it is technically possible the 11th or 25th hit, etc., could also be just as important. This limitation is true for essentially any large-scale unsupervised ranking algorithm. The exact queries to produce the results were provided in Section 2.3, and the free SemNet 2.0 source code link is available to readers as shown in the Data Availability Statement.

#### 4.4.2. Applied Limitations

Applied limitations include how the LBD algorithm can be utilized to interpret results of a specific inquiry. It should be noted that the intersection of the A5–A6 results examine direct connections to COVID-19. As such, the top-ranked intersecting A5–A6 concepts have known literature connections specifically to COVID-19. However, the algorithm cannot distinguish between symptomatic or asymptomatic illness or COVID-19 illness or symptom severity. Additionally, it does not distinguish between effects induced by natural COVID-19 infection versus a COVID-19 vaccination. Furthermore, the simulations cannot specify temporal order or temporal sequence of pathological relationships. Thus, the algorithm cannot differentiate between relationships that occur during active COVID-19 infection versus relationships that occur after COVID-19 recovery. Finally, there is a trade-off in known specificity of the ranked concepts’ relationship to the target nodes and attempting to identify lesser or under-studied relationships relevant to the target nodes. Simulations examining direct connections to the target nodes (e.g., intersection of A5–A6) will inherently be more specific but are more likely to produce well-known relationships. In contrast, cross-domain simulations with hub nodes (e.g., intersection of A1-A2-A3) are inherently less specific to the target nodes but enable the algorithm to predict under-studied concepts that could be just as important to the target(s) of interest. For this reason, it is important to report and interpret both direct simulation and cross-domain simulation results.

## 5. Conclusions

In conclusion, cross-domain text mining using artificial intelligence uncovered and prioritized connections between COVID-19 and resistant hypertension for future experimental assessment. While the ties to multi-scalar etiology were wide-reaching, there were a few consistent etiological themes synthesized: nodes that explained the entry or altered uptake of COVID-19 (dicer enzyme, VPS54); nodes related to inflammation or cytokine storm (tat genes, ATP6V0D1, TRIM40, gastrins, ribosomal protein S10); nodes involved in the cell cycle, DNA/RNA stability, or tumor/anti-tumor effects (haspin, ral guanine nucleotide exchange factor, BCR-ABL fusion gene, N-(3-oxododecanoyl)homoserine lactone, USP17L2, recQ4 helicase, HCF1, H3C2 gene, EXOSC2); nodes involved in lipid storage, metabolism, or atherosclerosis (choline phosphate cytidylyltransferase, MAP3K10, pegylated leptin, beta-lipotropin, CR 1392, high-density lipoprotein sphingomyelin, TPM2); nodes that alter sympathetic drive for blood pressure or involved in RAAS pathway (uridine kinase, metabotropic glutamate receptors, aspartic endopeptidases, MAP kinase kinase kinase 1, protein tyrosine phosphatase, cGMP-dependent protein kinase II, imipramine demethylase; and nodes that can modulate the endocrine system via the hypothalamic–pituitary–adrenal axis (somatotropin-releasing hormone, melanocyte-stimulating hormone, corticotropin, growth-hormone-releasing peptide-2, pro-opiomineralocorticoid, prolactin, des-n-octanoyl ghrelin, beta-lipotropin, gonadotropin, TAP-144 or liraglutide octreotide, somatotropin, and thyroid hormone).

## Figures and Tables

**Table 1:** Summary of analysis set-up using SemNet 2.0 to perform cross-domain text mining to elucidate relationships between COVID-19 and resistant hypertension. ID is the analysis name, Analysis Deliverable summarizes the analysis output, and Target Nodes with CUI describes the input of the analysis. Target nodes are the user-specified input into SemNet 2.0, which returns rankings for the related source nodes. Source nodes were limited to the following Unified Medical Language System (UMLS) node types: amino acids, peptides, and proteins (AAPP); hormones (HORM); and genes or genomes (GNGM). The listed target node CUI is the corresponding UMLS concept unique identifier.

ID	Analysis Deliverable	Target Node(s) with CUI
A6	Source nodes related to COVID-19	COVID-19 (C5203670)
A5	Source nodes related to resistant hypertension	Resistant hypertension (C0745130)
A4	Intersection of high-ranking A5 and A6 source nodes to determine hub nodes for cross-domain analysis in A1, A2, A3	COVID-19 (C5203670); resistant hypertension (C0745130)
A3	Relationship of AT1 receptor (a RAAS hub node) with COVID-19	AT1 receptor (C0529330); COVID-19 (C5203670)
A2	Relationships of epithelial sodium channel (a RAAS hub node) and COVID-19	Epithelial sodium channel (C0384156); COVID-19 (C5203670)
A1	Relationships of mineralocorticoid receptor (a RAAS hub node) with COVID-19	Mineralocorticoid receptor (C0066563); COVID-19 (C5203670)

**Table 2:** Summary of result metadata for the SemNet 2.0 simulations. Metadata includes source node count, metapath count, average HeteSim score, minimum HeteSim score and maximum HeteSim score. Metadata were used to normalize HeteSim scores and enable comparison and/or aggregation of simulation results. Note there are no metadata for A4 because it was not a separate simulation but, rather, the intersection of A5 and A6 filtered to obtain hub nodes used to construct the input for SemNet 2.0 simulations for A1, A2, A3.

ID	Target Node(s)	Source Node Count	Metapath Count	Average HeteSim	Minimum HeteSim	Maximum HeteSim
A6	COVID-19	75,731	41,932	0.2198	0.0001	1.0000
A5	Resistant hypertension	24,699	2883	0.3493	0.0003	1.0000
A3	AT1 receptor, COVID-19	67,779	69,722	0.2264	0.0002	1.0000
A2	Epithelial sodium channel, COVID-19	61,104	55,338	0.2091	0.0005	0.8292
A1	Mineralocorticoid receptor, COVID-19	70,038	77,486	0.2263	0.0002	1.0000

## Data Availability

SemNet 2.0 code can be found on GitHub https://github.com/pathologydynamics/semnet-2 (accessed on 9 August 2023).
